# Perception-action in children with ASD

**DOI:** 10.3389/fnint.2012.00115

**Published:** 2012-12-12

**Authors:** Claes von Hofsten, Kerstin Rosander

**Affiliations:** Department of Psychology, Uppsala UniversityUppsala, Sweden

**Keywords:** autism, perception, action, anticipation, planning, mirror-neurons, diffuse tensor imaging

## Abstract

How do disturbances to perception and action relate to the deficiencies expressed by children with autism? The ability to predict what is going to happen next is crucial for the construction of all actions and children develop these predictive abilities early in development. Children with autism, however, are deficient in the ability to foresee future events and to plan movements and movement sequences. They are also deficient in the understanding of other people's actions. This includes communicative actions as they are ultimately based on movements. Today there are two promising neurobiological interpretation of Autism Spectrum Disorder (ASD). First, there is strong evidence that the Mirror Neuron System (MNS) is impaired. As stated by this hypothesis, action production and action understanding are intimately related. Both these functions rely on predictive models of the sensory consequences of actions and depend on connectivity between the parietal and premotor areas. Secondly, action prediction is accomplished through a system that includes a loop from the posterior parietal cortex (PPC) through the cerebellum and back to the premotor and motor areas of the brain. Impairment of this loop is probably also part of the explanation of the prediction problems in children with ASD. Both the cortico-cerebellar loop and the MNS rely on distant neural connections. There are multiple evidence that such connections are weak in children with autism.

Perception, action and cognition are mutually dependent. Together they form functional systems, driven by motives, around which adaptive behavior develops (von Hofsten, 1993, 2004, 2007). Actions reflect all aspects of cognitive development including the motives of the child, the problems to be solved, and the constraints and possibilities of the child's body and sensory-motor system. Actions are directed into the future and their control is based on knowledge of what is going to happen next. Dysfunctions of the brain will affect the way subjects perceive the surroundings and how they organize their actions. Autism is a disorder in which the subject fails to attend to important varieties of social information and instead focuses on less informative physical aspects of the environment. In addition, actions are often compulsatory and stereotyped (see e.g., Bodfish et al., 2000; Goldman et al., 2008). Bodfish et al. (2000) found repetitive behaviors in both children with Autism Spectrum Disorder (ASD) and mentally retarded children but significantly more of them in children with autism. Furthermore, the prevalence of repetitive behavior, such as compulsion, was significantly correlated with the severity of ASD.

Deficiencies in the control of actions have not usually been considered to be core deficits of ASD or Asperger Syndrome (AS). Thus, the number of studies of action control in children with these syndromes is low compared to the studies focusing on the social aspects of the disorders. Recently, however, this picture is beginning to change. A number of studies focusing on action control in children with ASD have appeared. It is of great importance to identify the nature of the action problems associated with ASD, because this might provide crucial information for the understanding of what is failing in these children. Analyzing the physical movements has the potential of being helpful for objectively diagnose, treat and quantify performance gains starting at birth.

One widely used motor test is the Movement ABC (MABC-2) that includes a set of everyday action tasks such as walking on a line, putting beads on a string, standing on one leg, and throwing and catching objects. Green et al. (2002) used MABC-2 in a large, population-derived group of children. Definitive motor impairments were found in 79% of the children with ASD and a further 10% had borderline motor problems. Difficulty with the balance task in children with ASD stood out. In addition, the results show that children with ASD have greater difficulties in movement tasks that are both dependent on accuracy and timing, as seen in the timed peg-board tasks. Siaperas et al. (2012) tested 50 boys with AS and an equal number of typically developed boys between 7 and 14 years of age on MABC-2 and found that children with AS were especially deficient on the throwing and catching tasks, and the tasks on dexterity and balance. They also tested balance on one or both feet with open and closed eyes and found the children with AS and ASD to be deficient on all these tasks.

Although the general motor tests give clear indications of motor dysfunctions in children with ASD, they give less clear indications of what the specific problems are. From a perception-action perspective, the most important aspect of motor control is predictive control. Adaptive behavior has to deal with the fact that events precede the feedback signals about them. The only way to overcome this problem is to anticipate what is going to happen next and use that information to control one's behavior. There are many indications that children with autism are generally deficient in this kind of control.

## Postural control

Gravity is a potent force and when body equilibrium is disturbed, posture becomes quickly uncontrollable. Therefore, any reaction to a balance threat has to be very fast and automatic. Although several reflexes have been identified that help to control balance, postural reflexes are emergency reactions that tend to maintain balance at the cost of interrupting ongoing behavior. Disturbances to balance need to be handled by anticipating the upcoming problems and dealing with them in a predictive way.

Retrospective videos of children with autism indicate that postural control may be deficient already at an early age. For instance, Teitelbaum et al. (1998) showed a case of an 8.5-months-old boy who, when trying to maintain balance in a sitting position fell over “like a log” without using any allied reflexes to protect himself. In other cases they studied, the infant managed to sit for a few minutes at a time, but when the posture was asymmetrical as when reaching for objects or moving the arms and upper body, they fell over (Teitelbaum et al., 1998). Another less dramatic instance of poor postural control is the control of neck muscles when being pulled from a lying position. At 4 months of age an infant should be able to control his or her head position in this situation by maintaining it in line with the torso and not let it flop back. Flanagan et al. (2012) studied two groups of infants. In one group of 40 infants, all had older brothers or sisters with ASD. Ninety percent of those who went on to be diagnosed with ASD at 30–36 months had exhibited head lag at 6, 14, or 24 months. In another group of high-risk infants, Flanagan et al. (2012) tested for head lag at 6 months. They found that 15 out of 20 siblings of children who had been diagnosed with ASD exhibited head lag compared to 7 out of 21 of the low-risk siblings. Bhat et al. (2012) found that siblings of children with ASD also showed significantly more motor problems at 3 and 6 months of age compared to typically developing (TD) infants. In fact, the majority of the siblings showed both early motor delays and later communication delays.

## Anticipation

**Figure 1:**
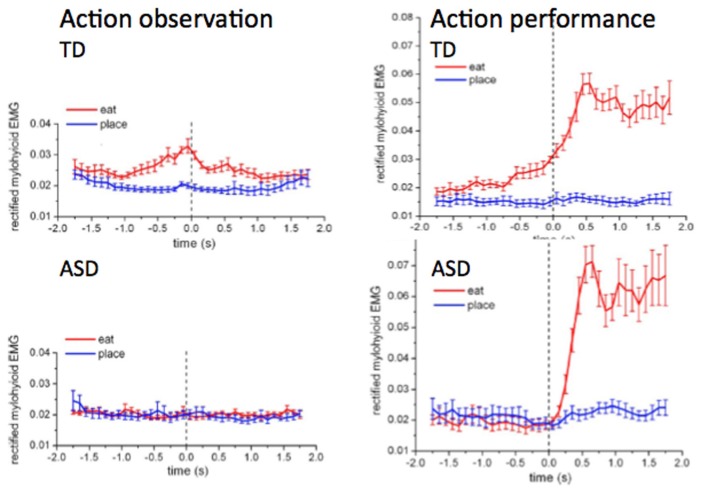
**The activation of the mylohyioid muscle during the event of bringing the food to the mouth (red) and the paper to the container (blue) in the TD children and the children with ASD.** Zero is the time of grasping. [From Cattaneo et al., PNAS, Copyright (2007) National Academy of Sciences, U.S.A].

There is evidence that children with ASD do not anticipate upcoming actions like TD children do. In a study of feeding, Cattaneo et al. (2007) measured activation of the mouth-opening mylohyoid (MH) muscle in 6–9-years-old TD children and children with ASD. The participants were asked to watch the experimenter performing two different actions: grasping with the right hand a piece of food placed on a touch-sensitive plate, bringing it into the mouth and eating it, or grasping a piece of paper placed on the same plate and putting it into a container, located on the experimenter's right shoulder. They found that children with autism did not show any activation in the MH when observing other people who brought food to their mouth, while TD children showed proactive activity in MH in this situation. This activity demonstrates that the TD children perceive other people's actions by activating their own action system in the way suggested by the Mirror Neuron System (MNS) hypothesis but that children with ASD don't. The result is shown in Figure 1.

A more remarkable result was that when, in a similar experiment, subjects brought food to their own mouth or a piece of paper to a container, strong pre-activation of the MH was obtained in the typically developed children about 1 s before the food arrived at the mouth, but in the children with autism the activation of the MH only started after the food was grasped (see Figure 1). Thus, the children with ASD did not chain the two actions together in a predictive way, i.e., they did not prepare the opening of the mouth before they brought food to it. To test whether this lack of chaining two actions is a more general phenomenon and not just confined to bringing food to the mouth, an experiment was also performed in which two other actions were performed sequentially. The tasks were bringing food or a piece of paper to two different containers. The container for the food was to be opened by the pressing a pedal by the foot while the container for the paper was already opened. Thus, the pressing of the pedal in the food case should be performed slightly before the hand arrived with the food. Predictive pressing of the pedal was performed by the TD children but not by the children with ASD. Anticipating the effects of one's own actions is an important aspect of motor control. In children with autism this ability seems to be impaired.

Further evidence for this hypothesis comes from a lifting task by Schmitz et al. (2003). They tested how children with autism and typically developed children (mean age 8 years) could maintain the left forearm stabilized in space despite imposed or voluntary unloading of a weight attached to it. In one condition a weight was attached to the left forearm arm with an electromagnet. The magnet was inactivated at a random moment and the weight then fell to the support. In the other condition the subjects unloaded the left forearm themselves by lifting the load with the right hand from a platform resting on the left forearm. EMG was recorded from the biceps and triceps brachii. It was found that the forearm stabilization in the loading condition was as equally good in children with autism and in typically developed children. The two groups differed, however, in the unloading condition. The latency of the biceps brachii inhibition for both groups was around 60 ms in the involuntary unloading situation. In the voluntary unloading condition, the latency for the ASD group was also 60 ms while there was a proactive activation by 15 ms for the typically developed children. This shows that the TD children anticipated the voluntary unloading while the children with autism did not.

Does this deficiency in motor control appear in development together with other signs of autism or does it precede them. A recent report on feeding indicates that deficient anticipation of actions is a precursor of autism (Brisson et al., 2011). Their study is based on retrospective analysis of family home movies. The results show that 4-months-old children who later become diagnosed with ASD anticipate less often the arrival of the spoon to their mouth in a feeding situation than do children who are not at risk. Anticipation was measured by counting the number of times the mouth failed to open before the spoon arrived.

## Planning movement sequences

Complex goal-directed movements are usually made up of several subunits that are chained together. When a movement is performed these subunits are linked in a predictive manner to create a continuous global action. Children with autism tend to split up such chained motor acts into unrelated movements. The result by Cattaneo et al. (2007) showing that these children do not link the approach of their hand to the mouth and the opening of the mouth is an example of such fragmentation.

To further test the hypothesis that children with ASD have a fragmented motor organization, Fabbri-Destro et al. (2009) asked children with ASD and TD children to execute two actions consisting each of three motor acts: to move the hand to and object, pick it up, and move to a container. The first two steps were identical but the last varied in difficulty. This was accomplished by varying the size of the opening of the container. The result showed that, unlike in TD children, the kinematics of the first motor act was not modulated by the task difficulty in children with autism. Similar results were obtained by Forti et al. (2011). They asked 12 high functioning preschool children with ASD and 12 TD children to grasp a ball, move it over the edge of a container, and drop it into a hole there. They found that while TD children did this in one continuous move, the children with ASD did it in a more discontinuous manner. First, they moved the ball to the container with no adjustment to what they were going to do next, then, in a discontinuous manner, turned the hand and dropped the ball. This resulted in more movement units and longer movement durations.

## Prospective looking

**Figure 2:**
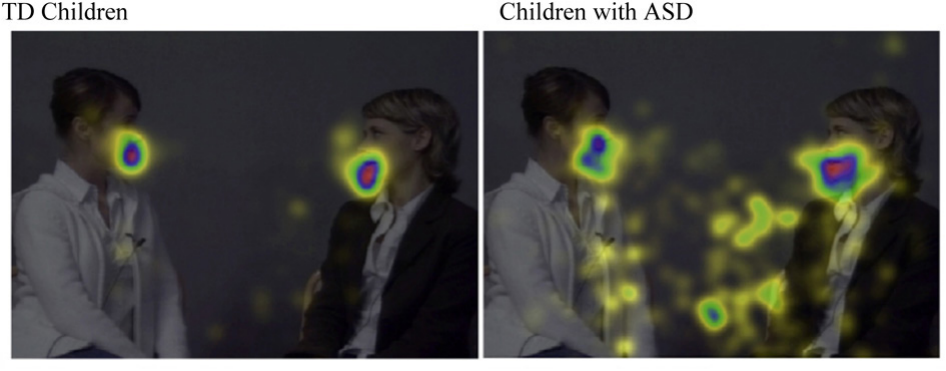
**The mean relative duration of fixations on the different parts of the display during the conversation.** The durations of fixation are calculated from a group of TD 3-years-old children (*n* = 12) and a group of 3–6-years-old children with ASD (*n* = 9). The increasing amount of fixations on a specific part of the display is depicted in yellow-green-blue-red color where red corresponds to the most fixations. It can be seen that the fixations of the children with ASD are more scattered than the fixations of the TD children. In addition, the fixation highlights show that the children with ASD have a strong tendency to fixate the shadow casted by the right model. When she spoke, she tended to move her head a little and the shadow of her head attracted the gaze of the children with autism but not the TD children (From von Hofsten et al., 2009, RASD, by permission).

Visual scanning the surrounding requires a plan for what to look at next. Children with ASD seem to be deficient in this ability. They do not look at the aspects of a social scene that are most informative (Klin et al., 2003) but instead fixate on insignificant details. It is not a question of being attracted by low-level visual saliency (Fletcher-Watson et al., 2009) or finding faces aversive to look at (Falck-Ytter and von Hofsten, 2011), but rather the lack of a plan of where to look next. von Hofsten et al. (2009) studied how TD preschool children and children with ASD look at when viewing a conversation between two models. They found that the TD children focused very much on the mouths of the talking models (around 70% of the time) while the children with ASD were much more scattered in their fixations and fixated the faces only 20% of the time. For instance, in contrast to the TD children they looked much at the shadow of the models. This is in line with Becchio et al. (2010) who found that instead of contributing to the perception of objects, shadows rather interfered with it. The fixation of shadows in children with autism is illustrated in Figure 2. An analysis was also performed on whether the subjects looked proactively at the next speaker of the conversation. It was found that this was the case in over 60% for the TD children while the children with ASD did it in less than 30% of the turns.

## Understanding one's own actions

Children with ASD have problems with representing their own actions. Several studies have used a specific test of this ability, (the Florida Apraxia Battery modified for children, Mostofsky et al., 2006). It consists of gestures to command, gestures to imitate, and gestures with tool use. The gestures to command and gestures to imitate include both transitive gestures (those that act on or with an object, e.g., hammering a nail) and intransitive gestures (those that do not act on or with an object, e.g., waving goodbye); the gesture for tool use section contains only transitive gestures. Dowell et al. (2009) found that children with ASD had worse basic motor skill and postural knowledge than did controls. The ASD group showed significantly poorer praxis than did controls after accounting for age, IQ, basic motor skill, and postural knowledge. Dyspraxia in autism therefore appears to be associated with impaired formation of spatial representations that are primarily visual in origin. MacNeil and Mostofsky (2012) investigated a group of children with ASD and a group with ADHD and found that whereas the children from both groups show impairments in basic motor control, impairments in performance and recognition of skilled motor gestures appear to be specific to autism. The specific impairment to represent one's own movements may also be related to deficiencies in imitation.

## The relationship to neuroscience

The children with ASD express problems that raises questions as to how they relate to brain processes. The fact that prediction of upcoming events is a major problem suggests that the cerebellum is involved. Haas et al. (1996) concluded from a review of 16 quantitative magnetic resonance imaging (MRI) and autopsy studies involving more than 240 autistic cases that cerebellar abnormalities are present in ASD from at least 5 years of age and throughout development. Cerebellar deficits could also explain why balance control is impaired (Dziuk et al., 2007). The posterior parietal cortex (PPC) has strong projections to the cerebellum via pons which then connects to the premotor and motor cortex. It has been suggested that this pathway is central for the prospective control of action (Altman and Bayer, 1997). It may also be one of the major routes for visuo-motor information to reach the premotor cortex and contribute to the evolving motor command (Miall, 2003). When this network is compromised, as it is in ASD, a number of prospective control problems emerge.

Another neural network associated with this ASD is the MNS (Oberman et al., 2008). It is primarily anchored in the Superior Temporal Sulcus (STS), the Inferior Parietal Lobule (IPL) and the Premotor Area (PA) (Rizzolatti and Craighero, 2004; Rizzolatti and Sinigaglia, 2010). Hence, the mirror cells in premotor cortex may code a motoric representation of visuo-motor actions, both during action execution and during action observation, driven by the cerebellar inverse model. It has been shown that MNS is compromised in children with ASD (Iacoboni and Dapretto, 2006) and that there is a significant negative relationship between degree of activation in Pars Opercularis (premotor area) as measured by fMRI and the severity of autism as measured by the social scale of the Autism Diagnostic Interview-Revised (ADI-R) (Dapretto et al., 2006). The activation of MNS is commonly studied by measuring activation in a specific frequency band in the EEG (the mu-rhythm corresponding to 9–13 Hz in adults). During rest the amplitude is high but during motor activity it decreases due to desynchronization of the activity. It also desynchronizes when subjects observe actions and that makes it reasonable to assume that the mu-rhythm reflect MNS activation. Oberman et al. (2005) found that the mu-rhythm in a group of ASD subjects desynchronized during their own activity but not during observation of other people's actions. This indicates that these subjects do not respond to other people's actions in the way they respond to their own. These findings were replicated by Martineau et al. (2008) giving support to the suggestion that the MNS system is deficient in children with ASD.

It has been suggested that the measured deficiency in the MNS in ASD subjects could be related to weak neural connections between IPL and PA (Mostofsky and Ewen, 2011). One thing that the trans-cerebellar loop and the MNS have in common is that both rely on long connections. It has therefore been suggested that long neural connections are weak in children with ASD and that shorter connections dominate, for instance those between the somatosensory cortex and the motor cortex (Haswell et al., 2009). To test this idea, Haswell et al. (2009) measured patterns of generalization in children who learned to control a novel tool and found that the children with autism formed representations that relied more than normally on association between motor commands and proprioception, that is between the neighboring areas of somatosensory cortex and M1. They also found that the greater the reliance on proprioception, the greater was the child's impairments in social function and imitation (Haswell et al., 2009). In TD children, action representations rely more on visual and auditory information that are defined in external coordinates.

Thus, the core problem in ASD may be more fundamental than the just an impairment of MNS. Both MNS and the trans-cerebellar pathway rely on long connections. Another set of long connections that are found to be weak in children with ASD, are the ones going through Corpus Callosum. The fact that they are weak in children with autism was discovered by diffusion tensor imaging (Alexander et al., 2007). The assumption of weak long-range connections in children with ASD is also supported by Wolff et al. (2012). They conducted diffusion tensor imaging on infants from 6 to 24 months of age and found that many of the long connections in the brain started off being overdeveloped in children who were later diagnosed with ASD but were clearly underdeveloped by 2.5 years.

## Conclusions

Although the most salient feature of Autism is a deficiency in communication and social ability, it is of great importance not to ignore the motor problems associated with ADS, because they might provide crucial information for the understanding of the dysfunction. It is clear that all communication consists of movements and that movement impairments give rise to disturbed communication, but it could not be the sole factor because many children with movement impairments are often good communicators. One important line of evidence suggests that children with autism are poor at predicting future events, at planning future actions and chaining action together. Prediction deficiencies are especially harmful when it comes to planning one's own actions and monitor other people's actions.

A recently discovered network in the brain, the MNS may be the mechanism that connects the motor problems and the social ones. According to the MNS hypothesis, observed actions are projected onto one's own action system together with the intentions and emotions associated with them. This facilitates the understanding of other people's actions. Impairments of the MNS will therefore have consequences both for social understanding as well as for the control of perception and action. If action planning is compromised, then the understanding of other people's actions will be compromised as well.

A number of conditions in the brain may be related to the evolution of autism but the most promising clue is the development of long connections between distant brain areas. Well-functioning long connections is crucial for the coordination of different brain areas and for the guidance of action by visual and auditory information. Impaired connections of this kind might be the ultimate reason why social function as well as prospective control are compromised in children with ASD.

### Conflict of interest statement

The authors declare that the research was conducted in the absence of any commercial or financial relationships that could be construed as a potential conflict of interest.
